# Micro/nanorobots for remediation of water resources and aquatic life

**DOI:** 10.3389/fbioe.2023.1312074

**Published:** 2023-11-09

**Authors:** Haocheng Wang, Yizhan Jing, Jiuzheng Yu, Bo Ma, Mingyang Sui, Yanhe Zhu, Lizhou Dai, Shimin Yu, Mu Li, Lin Wang

**Affiliations:** ^1^ State Key Laboratory of Robotics and System, Harbin Institute of Technology, Harbin, China; ^2^ Oil & Gas Technology Research Institute, PetroChina Changqing Oilfield Company, Xi’an, China; ^3^ State Engineering Laboratory of Exploration and Development of Low-Permeability Oil & Gas Field, Xi’an, China; ^4^ College of Engineering, Ocean University of China, Qingdao, China; ^5^ Department of Pharmacy, The Second Affiliated Hospital of Harbin Medical University, Harbin, China

**Keywords:** micro/nanorobot, water pollution control, water purification, actuation mechanism, decontamination mechanism, water monitoring

## Abstract

Nowadays, global water scarcity is becoming a pressing issue, and the discharge of various pollutants leads to the biological pollution of water bodies, which further leads to the poisoning of living organisms. Consequently, traditional water treatment methods are proving inadequate in addressing the growing demands of various industries. As an effective and eco-friendly water treatment method, micro/nanorobots is making significant advancements. Based on researches conducted between 2019 and 2023 in the field of water pollution using micro/nanorobots, this paper comprehensively reviews the development of micro/nanorobots in water pollution control from multiple perspectives, including propulsion methods, decontamination mechanisms, experimental techniques, and water monitoring. Furthermore, this paper highlights current challenges and provides insights into the future development of the industry, providing guidance on biological water pollution control.

## 1 Introduction

Water is a fundamental natural resource that is indispensable for biological survival, scientific progress and industrial development. In recent years, water resources have been continuously polluted, water scarcity has become a global problem. Some studies have shown that water pollution has not improved in those days, and there has been little actual progress in pollution prevention from a global perspective, with severe water pollution causing nearly 2 million deaths per year (Fuller et al., 2022). According to the United Nations Water Development Report 2023, there has been a consistent annual increase in water consumption over the past 40 years, and 10% of the world’s population is living in countries with high or severe water scarcity. Furthermore, droughts, overuse, anthropogenic pollution, political and geopolitical influences continue to cause a decrease in available water reserves (Gude, 2017), where human activities have become a major contributor to water pollution, with about 50% of anthropogenically generated wastewater being discharged directly into rivers or oceans without any treatment, resulting in serious ecosystem damages and long-term impacts (Shemer et al., 2023).

To conquer the increasing water pollution problem, various conventional purification methods have been employed, such as precipitation/encapsulation, adsorption, membrane technology, and more (Altowayti et al., 2022), these methods can achieve wastewater recycling and reuse through graded purification and sludge treatment. The precipitation/encapsulation method can convert soluble metal ions in wastewater into insoluble precipitate in a less costly way, but the generated sludge is easily affected by various types of oils and fats in the wastewater, which increases the difficulty of treatment. The adsorption method is mainly used for the treatment of organic pollutants in wastewater, the pollutants are stripped off by the adhesion generated at the interface of different phases. Adsorption is considered a low development and maintenance cost option (Abu Bakar et al., 2021), however, in the face of wastewater with more complex components, different adsorption layers are often required, which increases the construction cost in the implementation of the project. Membrane treatment is a relatively new water treatment method, which can extract both dissolved solutes and insoluble dispersed particles in water, targeting a wide range of pollutants and being very environmentally friendly (Yusuf et al., 2020). However, membrane treatment requires pre-treatment, which can otherwise damage the membrane (Rahman et al., 2023), this raises the cost of membrane treatment for further use. Although traditional water purification methods have been developed towards low-carbon and intelligent (Altowayti et al., 2022; Li et al., 2023), their disadvantages in terms of equipment construction costs, biocompatibility and recyclability limit their further development, especially in developing countries where they will not be able to be used in large quantities (Othman et al., 2022).

In recent years, micro/nanorobots and micro/nanomotor technologies have shown great advantages such as low cost, high efficiency and environmental friendliness in environmental remediation and water purification applications, which have gained widespread attention and have great potential for development and application. Micro/nanorobots (MNRs) or micro/nanomotors (MNMs), usually refer to microscopic substances with actuation capability between 1 and 1 mm in size, which can be both organic or inorganic, even artificially edited and modified microorganisms from nature. Micro/nanorobots convert external energy into self-propulsion through a variety of different physicochemical mechanisms (Urso et al., 2023), with common approaches including bubble (Liu et al., 2019; Ren et al., 2022; Yang et al., 2023), light (Xiong et al., 2022; Palacios-Corella et al., 2023), acoustic (Lu et al., 2020; Wang et al., 2021; Wang et al., 2022), magnetic (Li et al., 2017; Li et al., 2018; Sun et al., 2020; Yu et al., 2022; Li et al., 2023), biological (Lai et al., 2022) and other methods (Fu et al., 2019; Kong et al., 2019; Xu et al., 2019). In addition to a wide range of drive options, the working conditions for micro/nanorobots are much easier to achieve, some studies show that micro/nanorobots can even work under natural light (Kochergin et al., 2020; Ying et al., 2021; Ullattil and Pumera, 2023), without any pre-processing, obviously providing more possibilities in water control such as pollutants’ on-site instantaneous collection and degradation.

Furthermore, the self-propelled motion of the micro/nanorobots is often accompanied by the flow of surrounding fluids, eliminating the need for additional mechanical agitation required by traditional water pollution treatment methods and static nano decontamination techniques. This facilitates the process of pollutant capture and material transfer, leading to improved efficiency and reduced operation time in pollution treatment (Ying and Pumera, 2019). Additionally, micro/nanorobots can operate effectively in size-limited working conditions, especially within micro and nano-scale environments, which distinguishes them from traditional water pollution treatment methods. Based on these advantages, it is clear that micro/nanorobots are more in line with the principle of improving pollution treatment efficiency and expanding pollution treatment capacity and reducing pollution treatment cost (Raha et al., 2008). They also align with the direction of next-generation advanced water treatment technology, featuring real-time degradation, recycling, reuse, and higher material utilization rates.

Based on the studies conducted between 2019 and 2023, this paper provides an overview of micro/nanorobots’ role in the field of water pollution control. Here the driving mechanisms and methods, specific decontamination methods, targeted pollutants, experimental technologies and platforms have been reviewed. This paper also discusses the feasibility and necessity of micro/nanorobots used for long-term water monitoring. Analysing principles and presenting cases, this paper describes the relevant problems currently faced and envisions the future development of the industry.

## 2 The actuation of micro/nanorobots

### 2.1 Actuation mechanisms

The actuation of micro/nanorobots is very different from that of macro-robots, the former cannot achieve effective motion through inertial force, and the “scallop theory” proposed by Pursell in 1976 well explained that time-symmetric motions of an object in low Reynolds number environments cannot result in displacement (Zhou et al., 2021a). Therefore, the actuation of micro/nanorobots is different from the “brute force actuation” of macroscopic objects and is based on a new kinematic mechanism that utilizes gradients for actuation. In fact, the motion of all micro/nanorobots is the motion from high gradient positions to low gradient positions, and all enhancement of the motion effect is the enhancement of the gradient, including a larger gradient difference, or a higher efficiency of the gradient utilization. The specific method of generating gradients is to generate asymmetry, which is done in the design process of micro/nanorobots through the selection of materials or the design of structures. Common methods to generate asymmetry include the use of magnetic fields, acoustic fields, electric fields, concentrations of chemicals, and the asymmetric structures of robots, *etc.* (Wang and Zhang, 2021).

### 2.2 Actuation methods

#### 2.2.1 Bubble-driven propulsion

Bubble-driven propulsion is one of the most common methods for actuation. When micro/nanorobots move in the water, various biochemical reactions or physical phenomena on their surfaces lead to the generation of micro/nano-sized bubbles. These bubbles accumulate at the back of the micro/nanorobots and propel them forward by moving in the opposite direction. Depending on whether the body of the micro/nanorobot serves as a consumed substrate or not, bubble-driven micro/nanorobots can be categorized into two types: “catalytic fuel decomposition” and “self-decomposition”.

**FIGURE 1:**
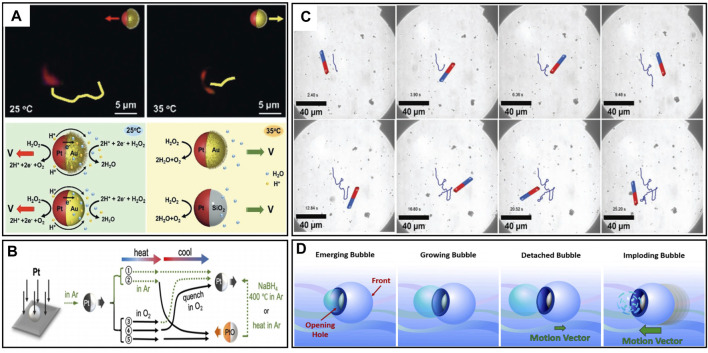
**(A)** Diagram of the actual movements and the surface actions of the PNIPAM@Au–Pt micromotors in the Pt direction and Au direction (Ji et al., 2019). **(B)** Varying compositions of the micromotor through five different manufacturing paths to achieve different movement effects (Lyu et al., 2022). **(C)** Motion trajectories of FeOx@MnO_2_@SiO_2_ micromotors at 1 wt% of H_2_O_2_ solution guided by a magnet (Yang et al., 2023). **(D)** Mechanism diagram for the four steps of the bubble implosion propulsion (Zhao et al., 2022).

Catalytic fuel decomposition robots contain a catalyst for fuel decomposition reactions. For example, a robot might incorporate asymmetrically distributed Pt metal, which catalyses the decomposition of gas bubbles in the presence of H_2_O_2_, and the robot itself does not experience any loss during the movement process. Ji et al. (2019) designed a bimetallic Janus micromotor PNIPAM@Au-Pt with bidirectional movement capability. The Pt part can be oriented to serve as either the front or back, allowing versatile movement. The driving mechanism involved Pt-catalyzed H_2_O_2_ decomposition, resulted in self-diffusion for movement. Temperature-induced PNIPAM chain modifications controlled the micromotor’s hydrophilic and hydrophobic functions. At temperatures below 32°C, electrons and protons migrated from the Pt region to the Au region and reacted with H_2_O_2_ to generate oxygen, when the temperature surpassed the critical point, a phase transition occurred, changing the hemisphere of the micromotor in contact with the fuel, switched propulsion mechanism from self-electrophoretic propulsion to self-diffusive propulsion. The actual movements and the different surface reactions of the PNIPAM@Au–Pt micromotors are shown in Figure 1A. The significance of this study lies in achieving micro/nanorobots steering through thermal control, offering valuable insights for subsequent module design.

A recent study has shown that the generation of intermediate platinum oxides also affects the actuation of micro/nanorobots during Pt-catalyzed H_2_O_2_ decomposition. Lyu et al. (2022) observed a distinct behavior on conventional Pt-SiO_2_ robots, their robots moved towards the Pt layer instead of away from it when the thickness of the Pt layer exceeded 100 nm. After excluding possible variables such as temperature, they determined that the excessive thickness of the Pt layer and the presence of platinum oxides, mainly PtO, induced a reversal in the direction of motion through an auto-electrophoretic effect. This discovery enables precise control over the motion direction of micro/nanorobots by controlling the Pt oxides. This breakthrough introduces new materials for the motion control of micro/nanorobots. Five paths for manufacturing are shown in Figure 1B.

Using expensive Pt for micro/nanorobot fabrication can lead to high production costs. Furthermore, the susceptibility to electrochemical corrosion from Sulphur-containing substances and other metal ions can degrade its catalytic performance and cause the failure of the robots, known as “Pt-poison” (Casado-Rivera et al., 2003; Orozco et al., 2013; Zhao et al., 2013). To address this, researchers have explored alternative catalytic materials like manganese oxides, such as MnO_2_ (Yang et al., 2023), Mn_2_O_3_ (Li et al., 2021), and Mn_3_O_4_ (Bing et al., 2020), among which MnO_2_ is most widely used. Manganese dioxide shows the advantages of abundant sources, low cost, and stable chemical properties. It can simultaneously participate in the oxidation process of pollutants and is commonly employed in the treatment of organic pollutants. Yang et al. (2023) designed a multifunctional FeO_x_@MnO_2_@SiO_2_ microrobot capable of degrading antibiotics such as Naproxen in a peroxymonosulfate (PMS)/H_2_O_2_ system. In this robot, MnO_2_ is responsible for decomposing H_2_O_2_ into oxygen bubbles, providing propulsion in the solution and generating substances for antibiotic degradation. The trajectories of a robot guided by ais shown in Figure 1C. Through experiments of varying durations, they explored the connections between robot structure, velocity, and degradation efficiency, revealing the relationship between catalytic efficiency, motility behavior, and the mechanism of motion. A highlight of their work is that PMS can regulate the excessive formation of O_2_ resulting from H_2_O_2_ decomposition by occupying the Mn site through interactions with the robots.

Self-decomposing micro/nanorobots directly engage in reactions as substrates. For instance, a robot containing Mg metal catalyzes H_2_O_2_ decomposition in an acidic environment. The endurance matches the Mg consumption time, rendering the robot immobile after the Mg depletion. Zhao et al. (2022) designed a fully biodegradable microrobot propelled by hydrogen bubbles from the Mg-hydrochloric acid reaction. The mechanism diagram is shown in Figure 1D. Investigating the impact of bubble stabilizers on propulsion patterns, they tested hydrochloric acid (six concentrations) and surfactant Triton X-100 (five concentrations). Findings demonstrated surfactant influence on the propulsion direction of the robot. Generally, bubble-driven micro/nanorobots exhibit lower motion precision than methods like magnetic driving. For self-decomposition robots, bubbles are randomly generated, producing disordered motion with limited control and switching capabilities. The very limited endurance and the potential for secondary contamination (e.g., Mg^2+^) render self-decomposition micro/nanorobots incapable of performing tasks with high precision.

Both of these bubble-driven propulsion methods rely on fuel decomposition. However, suitable fuel chemicals are often lacking in polluted water, leading to the need for artificial addition of chemical reagents like H_2_O_2_. The introduction of H_2_O_2_ has both advantages and disadvantages. On one hand, its role as a strong oxidizing agent enhances the pollutant oxidation process, contributing significantly to pollutant degradation. Conversely, the addition of hydrogen peroxide itself is costly and the addition of additional strong oxidizing agents to an open body of water can cause unwanted secondary damage to the body of water. It is noted that bubbles themselves can function as microscopic robots. Generated bubbles, often at the micro/nano-scale, come with physical adsorption effects on their surface. The presence of microbubbles or clusters can further amplify the efficiency of water pollution treatment.

#### 2.2.2 Light-driven propulsion

Light-driven propulsion has become a popular option for water pollution treatment due to the ready availability of natural light energy. Additionally, light-driven materials tend to generate reactive oxygen species (ROS) through the photo-Fenton reaction, enhancing the treatment efficiency of pollutants (see Section 3.1 for more details). With the deepening of the study, new light-responsive materials are applied, more specific parameters are investigated, and new light-triggered motion mechanisms are discovered.

The use of light-driven micro/nanorobots converts light energy into kinetic energy, utilizing various frequency bands of light. The more commonly used bands include visible light, near-infrared light, and ultraviolet light. However, light cannot drive all forms of matter. Photoactive materials, such as semiconductors, are necessary to create a net displacement under light.

**FIGURE 2:**
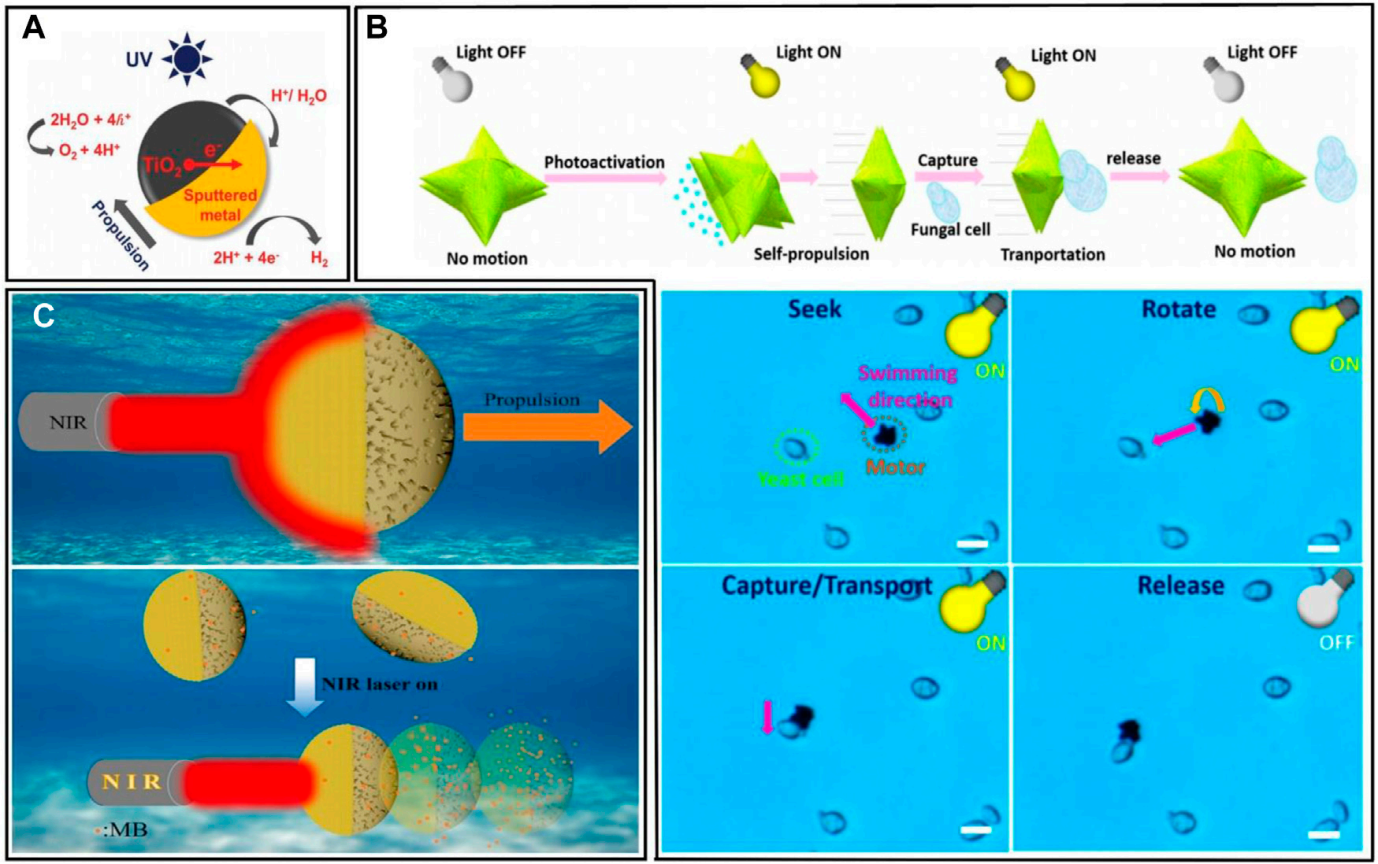
**(A)** Schematic illustration of the reactions of the metal/TiO_2_ micromotors under UV light irradiation in study (Maric et al., 2020). **(B)** Sketch of the workflow of BiVO_4_ micromotors for microorganisms’ removal, and photos of BiVO_4_ micromotors capturing microorganisms under UV light irradiation (Villa et al., 2019). **(C)** Scheme of NIR light-propelled motion for MB removal. This microrobot made a 10.2% increase in the removal efficiency of MB dye under NIR light irradiation (Li et al., 2023).

TiO_2_ is a commonly used semiconductor in the fabrication of micro/nanorobots. Not only does TiO_2_ have a programmable surface charge, providing the ability to specifically identify contaminants, but it also has a Schottky barrier at its interface, which would result in higher photocatalytic activity. The common use of TiO_2_ is with the combination of different metals. In fact, among the many metal-TiO_2_ combinations, platinum is the most effective and provides the most stable power (Maric et al., 2020). One common surface reaction of the metal/TiO_2_ robots is shown in Figure 2A.

Functional optimization based on TiO_2_ robots is a major research direction in the field. Xiao et al. (2020) tripled the motion speed of a conventional TiO_2_ robot by adding an Au/Ag bilayer coating. Through their experiments, they ruled out the possibility that the Ag in the bimetallic layer alone could lead to the speed increase and determined that the metallic coating works through synergy to increase the speed of the TiO_2_ robot in the light. This synergistic action did not depend on any fuel, and the kinematic efficacy enhancement was achieved even in pure water.

Except for TiO_2_, other materials are also applied in the manufacturing of micro/nanorobots. Villa et al. (2019) designed a micro/nanorobot using the photoactive substance BiVO_4_ to capture microorganisms. Under visible light, this robot can actively seek out and non-destructively adhere to yeast cell walls through linear motion, allowing for controlled capture and release of yeast cells by switching the visible light on and off, which can serve as disinfection motile tools for the removal of microbial contamination in water (Figure 2B). Li et al. (2023) constructed near-infrared light-driven micro/nanorobots named JMPPs@Au by depositing Au nanoparticles (NPs) onto the half surface of MPPs. The motion propelled by NIR light is explained in Figure 2C. By controlling the light on and off, their experiments demonstrated the positive impact of light on pollution removal.

These materials absorb photon energy, generating electron-hole pairs (e^−^-h^+^) exposed to light. When excess electrons accumulate on the surface of the material, they can react with pure water, generating bubbles and initiating a photochemical reaction under light irradiation. This process ultimately leads to bubble creation or autophoresis, showing another major advantage of certain light-driven robots, which is their independence from external fuel sources (Urso et al., 2023). Wang et al. (2020) designed a cost-effective, easily fabricated robot with good motion capabilities even under low light intensity. The robot did not rely on any fuel source operating across the entire visible light spectrum. It achieved impressive movement, covering 18 times its body length in just 1 s in pure water. Even at one-third the intensity of sunlight it showed great motion effect. This study presents the fastest pure water-driven micro/nanorobots and highlights the advantages of non-toxicity and minimal secondary pollution.

**FIGURE 3:**
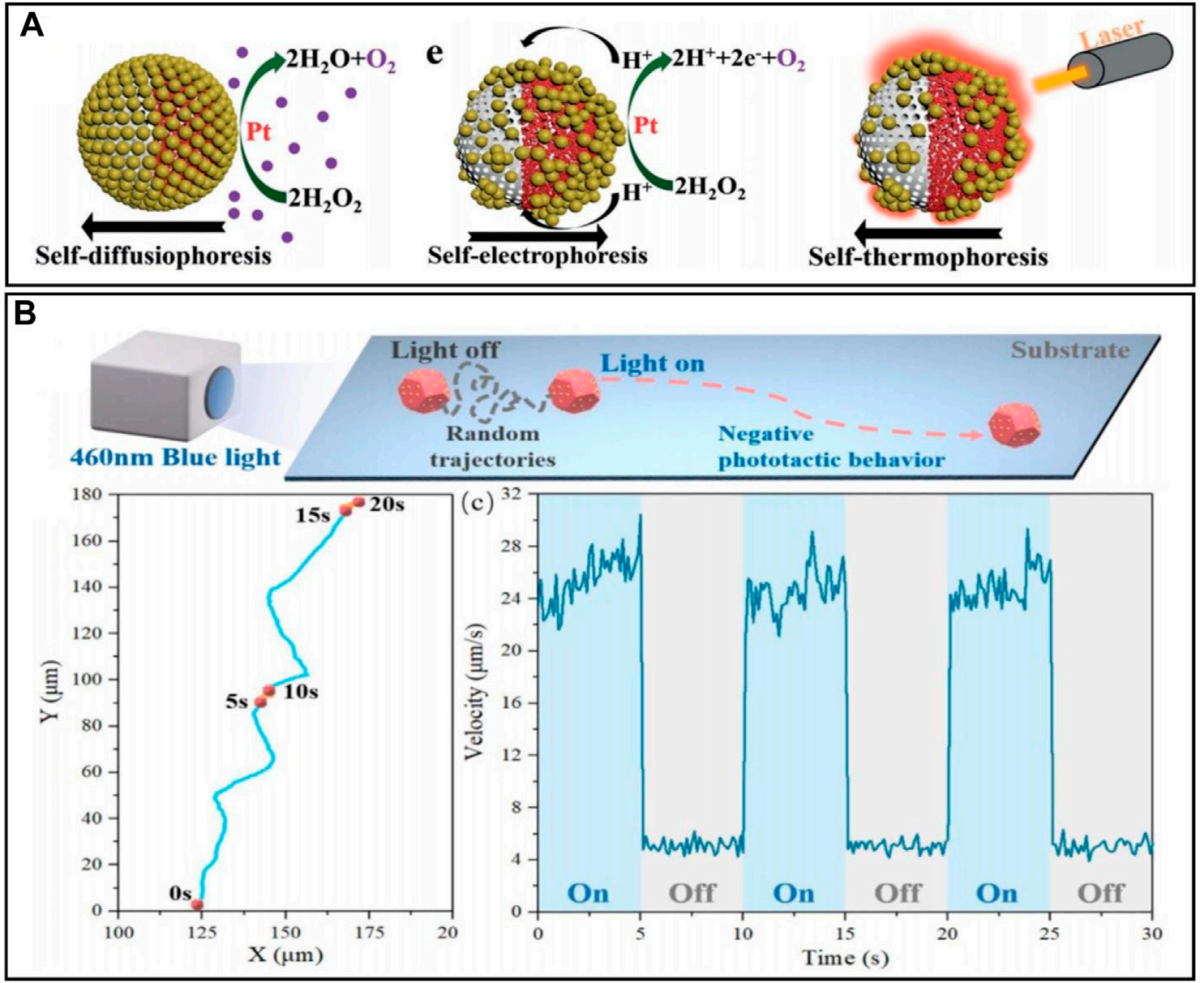
**(A)** Schematic illustration of the propulsion mechanism, switched self-electrophoretic propulsion, light-induced self-thermophoretic propulsion (Xing et al., 2020). **(B)** Schematic illustration of light-controlled self-propulsion behavior of Cu_2_O@CdSe micromotors. Trajectories and the speed of the Cu_2_O@CdSe micromotors changed as the light switched on and off. Experimental conditions: 0.005 mM tannic acid solution and 1.8 W/cm^2^ blue light (Chen et al., 2021).

Since the robot can be activated by all light larger than the material band gap, this represents a large range of light that can open up light-driven micro/nanorobots, which provides the possibility of controllable multimodal motions. Xing et al. (2020) reported a micro/nanorobot with pH-responsive multilight propulsion. The surface reactions of the robot are shown in Figure 3A. The robot was first propelled by self-diffusion in 3.0% hydrogen peroxide. Then, the weak acidity of H_2_O_2_ triggered the disassembly and reorganization of gold nanoparticles on the robot, leading to a contact between gold and platinum nanoparticles, which changed the propulsion mechanism to self-electrophoresis. Asymmetric and aggregated gold nanoparticles can also generate a thermal gradient under laser irradiation, thus generating motion by auto-electrophoresis. The robot can achieve three different mechanisms of optical propulsion. Chen et al. (2021) designed a Cu_2_O@CdSe micro/nanorobot driven by visible light, which is able to move horizontally or vertically by only adjusting the direction of the asymmetric light field, and the stopping and going can be controlled by switching on and off the light (Figure 3B). This design accomplished multidirectional positioning of the robot. Feng et al. (2021) designed a robot with more complex functions, and their robot can achieve orientation control and vertical motion, horizontal motion, and rotation.

With easier-to-create driving environment, more readily available driving fuel, fast robot movement speed, wide range of target pollutants, and beneficial concomitant reactions such as the photo-Fenton reaction, light-driven micro/nanorobots have demonstrated great advantages for applications in water pollution treatment.

#### 2.2.3 Magnetic actuation

Magnetic actuation presents another prevalent option. A micro/nanorobot with magnetic abilities exhibits motion that consumes minimal energy in an inhomogeneous magnetic field, where the asymmetry primarily arises from the magnetic field gradient. In contrast to various other drive methods, magnetic drives can provide very high accuracy, which is advantageous when performing precision operations (Ji et al., 2021; Ji et al., 2023). Meanwhile, the main drawbacks of magnetically driven micro/nanorobots are that the mechanical structure is relatively fixed and typically requires stronger actuation forces to overcome friction from the substrate or surrounding fluids (Hou et al., 2023a).

The magnetism of a micro/nanorobot is designed by adding magnetizing materials to the robot. Common materials include Ni, Fe_2_O_3_, Fe_3_O_4_, *etc.* The use of these materials provides modularity to the robot design, which can be referred to as a “magnetic drive module”. A magnetically propelled sponge porous microrobot is presented by Ma and wang (2019). This robot possessed super-hydrophobicity and achieved *in situ* oil removal. They enhanced a commercially available sponge material by incorporating superhydrophobic PDA/Fe_3_O_4_ nanoparticles, resulting in the creation of a three-dimensional porous superhydrophobic sponge with magnetic properties, which can be used to effectively adsorb various types of oil contaminations. They also conducted experiments in path planning, enabling the robot to follow different routes for removing oil droplets from water. This demonstrated precise motion control throughout the operation. The sponge robot maintained a high absorption capacity even after five reuses, providing a reference for the potential reuse of superhydrophobic sponges for environmental remediation.

**FIGURE 4:**
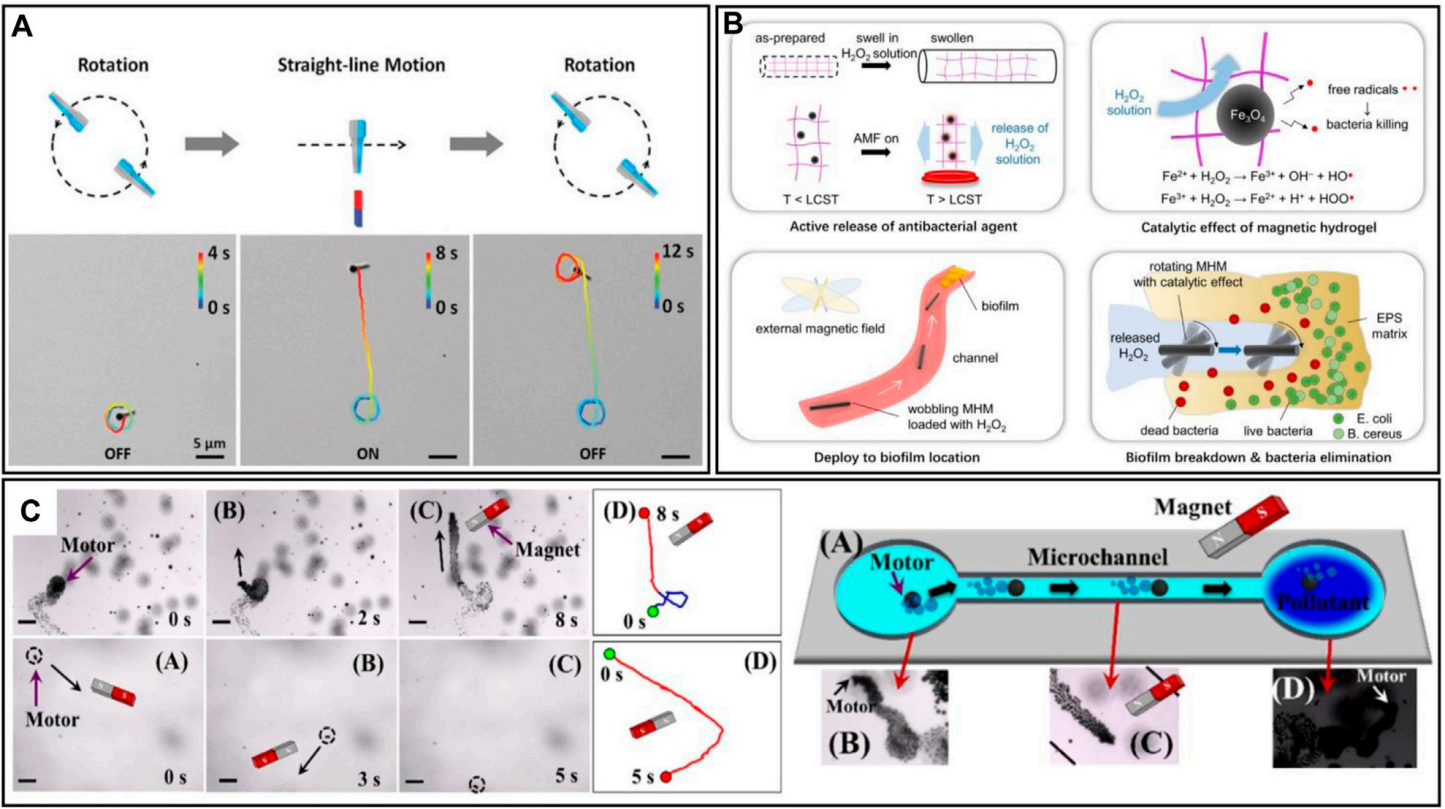
**(A)** The motion trajectory of a magnetically guided micromotor move in H_2_O_2_ fuel and pure water. The Figure also shows a micromotor traps contaminants in the microchannel, and the real-time images of the process (Singh et al., 2020). **(B)** Schematic illustration of cargo release when heated above the lower critical solution temperature (LCST) of the motors; bacteria-killing free radicals generated in the process of Fenton reaction when the motors are immersed in the H_2_O_2_ solution; a wobbling motion of the motor driven by the external magnetic field; disruption on the biofilm by catalyzing the released H_2_O_2_ solution (Sun et al., 2023). **(C)** Schematic illustration of the motion of the magnetic matchstick micromotor in the magnetic field. The Figure also shows trajectories of the magnetic matchstick micromotor moving in 2% H_2_O_2_ solution when the magnetic field is on or off (Zhang et al., 2021).

In fact, the incorporation of magnetically driven materials into robots has gained widespread acceptance, even in challenging environments where magnetic propulsion is difficult to achieve, such as open water. Additionally, in operational contexts that do not require delicate manipulation, researchers still incorporate magnetic materials where appropriate, primarily to harness the magnetic properties for recycling purposes. The study by Singh et al. (2020) also highlights the magnetic recyclability of micro/nanorobots. Their micro/nanorobot was developed using carbon nanospheres with Pt for H_2_O_2_ decomposition (propulsion) and Ni for magnetic attraction (magnetically driven module). The robot was specifically designed for dye degradation and cannot degrade oil droplet material in real-time. Therefore, after operating in an oil-contaminated environment, the retrieval of both the contaminants and the micro/nanorobot is necessary. As shown in Figure 4A, tasks can be easily accomplished using magnets.

Furthermore, there are examples of micro/nanorobots exhibiting multimodal motions under magnetic field actuation. Sun et al. (2023) developed a magnetic hydrogel micro/nanorobot (MHM) capable of mechanically disrupting biofilms. The work of the robot can be found in Figure 4B. By incorporating Fe_3_O_4_ material, the robot responds to an external magnetic field and utilizes Fe_3_O_4_ for contaminant treatment through the Fenton reaction. The MHM operated in two modes of motion controlled by an applied magnetic field, planar rotational and oscillatory modes. The study noted that the robot can be loaded with H_2_O_2_, which in this case, served as a biocide rather than a motion fuel. Zhang et al. (2021) designed a match-shaped micro/nanorobot with two modes of motion, rotary motion and linear motion under a magnetic field (Figure 4C). By switching the external magnetic field, they can realize the *in situ* switching of motion modes. This study is promising for micro-motion and sensing applications. In addition, it has been shown in the literature that liquid-based reconfigurable microrobots can offer additional possibilities under magnetic control. Ferromagnetic fluids have different motion and deformation behaviours, e.g., they can split/aggregate, or transform states. In addition, the self-assembly and collective behaviour of ferromagnetic fluids show excellent motions, which may show more applications in the field of water pollution treatment in the future (Wang et al., 2023a).

Clusters of micro/nanorobots can be used to compensate for the shortcomings of individual robot motion. Various approaches such as parallel operation and coordinated operation on multiple objects have been proposed, as well as advanced drive mechanisms and operation schemes (Wang et al., 2023b). However, trying to make clusters that are in the same environment exhibit different responses is currently subject to further research. Xu et al. (2022) proposed a new decoupled control method to achieve different movements for micro/nanorobots in the same magnetic field. They designed the micro/nanorobots with different magnetization directions to perform phase-different planar crawling motions within an oscillating magnetic field. Based on this model, complex co-operation of micro/nanorobots under the same excitation will become possible.

#### 2.2.4 Biological actuation

Biological actuation refers to the modification of natural microorganisms into robots through surface modifications and the addition of functional modules. Unlike the previously mentioned drives, which are externally controlled, this category represents truly self-driven micro/nanorobots. As microorganisms are natural materials, the utilization of bio-driven micro/nanorobots offers viable ways to mitigate the risk of potential secondary contamination.

**FIGURE 5:**
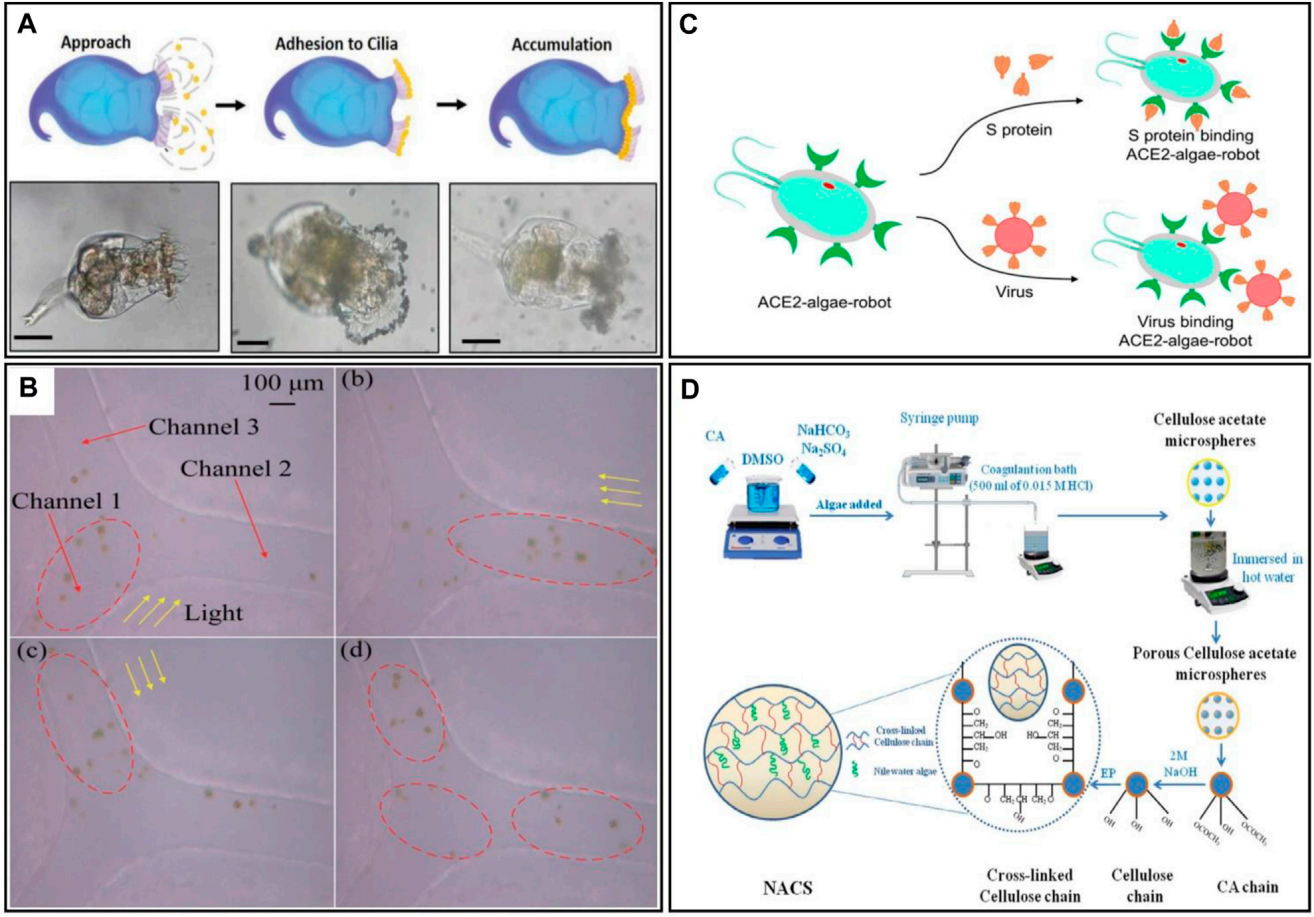
**(A)** Schematic illustration of the Mechanism for the formation of the rotibot, and the microscopy images of the Mechanism for the formation of the rotibot (Soto et al., 2019). **(B)** Images of mass *E. elegans* cells swimming in the Y-shaped microfluidic channel. Algal cells are in the red ellipses, yellow arrows represent light direction (Xie et al., 2021). **(C)** Schematic illustration of the ACE2-algae-robot remove the spike protein and SARS-CoV-2 virus (Zhang et al., 2021). **(D)** Schematic illustration of the fabrication of the Nile water algae cross-linked cellulose microsphere (NACS) (Moghazy et al., 2023).

Soto et al. (2019) designed a rotifer microrobot, named “rotibot”, by utilizing a marine rotifer as a carrier (Figure 5A). The rotifer was electrostatically attached inside the mouth by functionalized microbeads. The movement of the cilia of the rotifer created fluid flow toward its mouth, enabling the treatment of contaminants through functionalized microspheres. The robot relied exclusively on the self-propulsion of the rotifers and did not require any external fuel. Through experiments, the robot demonstrated its effectiveness as a dynamic micro-cleaning platform for the removal of a variety of environmental contaminants, including *E. coli*, neurotoxicants, and different heavy metals. Additionally, Shimizu et al. (2021) achieved the design of a biohybrid windmill by placing captured microalgae within an orthogonal frame. By incorporating capture chambers at the edges of the frame, they constrained the motion of the captured microalgae to be isotropic to the orthogonal axes. This design aimed to prevent motion cancellation caused by the non-uniform movement of the microalgae. Experimental tests demonstrated that the bio-windmill could rotate at an angular velocity of 0.78 rad/s. Similar to this work, Xie et al. (2021) achieved the manipulation of artificial microstructures through algal cells by capturing algal cells in tiny structures. The orientation of the robot can be controlled by capitalizing on the phototropism of the algae with the integration of an external light source. The motion of the robot when entering the Y-shaped tunnel is shown in Figure 5B. This study successfully demonstrated the cargo transport and autorotation capabilities of an algal microrobot, showing its potential for precise object manipulation on a miniature scale.

In addition to the aforementioned examples, algae have also been used in the assembly of other types of robots. Zhang et al. (2021) developed algal microrobots that can be used to remove the SARS-CoV2 virus from wastewater by modifying the surface of algae with ACE2 receptors (Figure 5C). ACE2 receptors, known for their high affinity to viral spiking proteins, serve as potent cellular receptors for SARS-CoV-2, enabling targeted virus recognition. The researchers selected Chlamydomonas reinhardtii due to its ease of mass production, swift mobility in various water environments, extended lifespan, and feasibility for surface modifications. Their experimental results demonstrated an impressive 89% removal rate of SARSCoV-2 pseudo-virus, providing a design concept and template for real-time purification of coronavirus wastewater. Similarly, Moghazy et al. (2023) employed Nile algae to create porous cellulose microspheres designed for the removal of methylene blue dye and copper ions from aqueous media, significantly enhancing the adsorption capacity of their robots. The fabrication of their robot is illustrated in Figure 5D.

#### 2.2.5 Other actuation methods

Besides the above methods, there are also common actuation methods such as the Marangoni effect, thermal-driven propulsion, acoustic actuation, optimal actuation and electric manipulation. More specifically, techniques like laser scanning and even photosynthesis have been utilized.

The Marangoni effect is the phenomenon of mass transfer due to the existence of a surface tension gradient at the interface of two liquids with different surface tensions. This effect can be triggered by various mechanisms. For example, Sun et al. (2019) designed a novel micro/nanorobot with controllable motion behavior based on infrared light. The propulsion of this robot relied on the Marangoni effect generated when polypyrrole nanoparticles (PPyNPs) were exposed to infrared light. PPyNPs exhibit strong absorption bands in the near-infrared region, allowing them to convert light energy into heat. This process reduced the local surface tension near the irradiated area, leading to the generation of a solution tension gradient. The cessation of the robot’s motion can be achieved by adding surfactants to eliminate the thermal Marangoni effect, counteracting most of the tension gradient. Another classic example is the robot designed by Yoshida et al. (2021) for release via ethanol, which also serves as a classic design for energy storage. During operation, the loaded ethanol diffused spontaneously. The released ethanol rises to the free surface due to buoyancy and reduces the surface tension of the water, created a tension gradient that enables the robot to move.

Ultrasonic and electric fields are also significant driving methods for micro/nanorobots. Xiaolong Lu et al. designed a device based on locally enhanced acoustic flow for driving microrobots. The platform conducted precisely transport along a given path. Through numerical simulations and specific experiments, it was demonstrated that the acoustically driven microrobots have high motion accuracy (Lu et al., 2019). Reorientation of the water dipole moment due to a rotating electric field can rotate carbon nanotubes immersed in aqueous solution, which gives theoretical basis for electric manipulation of underwater micro/nanorobots. The study of Zhongyu Fu et al. showed that the radius and length of the nanorobot affects the rotation angle, speed and cycle time, providing a reference for the use of micro/nanorobots underwater guided by electric fields (Fu et al., 2019).

A classic case of thermal drive is the thermal fluctuation-based motor designed by Lou et al. (2021) utilizing inherent thermal motion. Their self-thermal reflective nanorobot, built on Janus particles with unidirectional transmission, can reverse direction by adjusting the frequency of an external potential. The motor achieved a speed close to the superposition of the drift speed of a pure Brownian motor and the self-propulsion speed of a pure auto-thermophoretic particle. Furthermore, Kim et al. (2019) designed a laser scanning-based micro/nanorobot using shape memory alloy (SMA). Each laser scan triggered a material deformation, enabling the crawling motion of the robot. The wireless self-recovery-like motion achieved by this robot can serve as a reference for other deformable micro-scale structural driving methods.

#### 2.2.6 Enhancement of drive effects

The enhancement of micro/nanorobot motion effects through algorithm optimization is also a mainstream direction. Algorithms and various optimized control methods can be used to compensate for design flaws in the robot body and improve motion performance. Hou et al. (2023b) designed a microdrill that enabled propulsion driven by a magnetic field. By introducing a camera system, they implemented a control strategy that can utilize vision for dynamic feedback, thereby switching the direction and frequency of rotation according to the local environment, enabling the microdrill to move flexibly and penetrate obstacles efficiently. Xu et al. (2022) developed a broad learning system (BLS) based learning servo control strategy and applied it to a micro-robotic system. They combined the traditional Lyapunov theory with a new learning-based approach to obtain constraints on the controller parameters. Their model did not require adjusting of the controller parameters and achieved an applied extension of BLS. Liu et al. (2021) proposed a path planning algorithm for three-dimensional motion of micro/nanorobots. Their design can compensate for the angle between the robot’s direction and the magnetic direction when there are weight perturbations and lateral perturbations in 3D space. Experiments demonstrated that their method has sub-millimeter accuracy in three-dimensional space.

Through the design and integration of modules, it is possible to achieve the effect of “1 + 1>2”. Zheng et al. (2021) designed a hand module that can grip tiny objects by changing the local ion density or pH. Through experiments, they performed grasping and transport of magnetic microspheres, demonstrating the effectiveness of the design. This programmable hand-like structure offers possibilities for the dynamics of micro-nano robots. Zheng et al. (2022) also designed a single-step aniso-electrodeposition method that can be used to fabricate modular micro/nanorobots. Such modular robots can achieve a wide range of motions such as spiralling, twisting, bending and curling, and perform multiple tasks such as propulsion, grasping and object transfer simultaneously in response to magnetic field, ionic and pH stimuli. In addition, the authors further explored the possibility of loading other functional modules (e.g., cells, drugs, and magnetic nanoparticles) onto this module to achieve multifunctionality.

In addition, hybrid actuation has been developed through module integration in the design of micro/nanorobots (Wang et al., 2020). In the field of water pollution, the main focus is on hybrid designs between optical, bubble and magnetic drives (Ying et al., 2019; Shen et al., 2020; Li et al., 2022; Mayorga-Burrezo et al., 2023). Through the hybrid-driven operation, the operation accuracy can be improved, the operation range can be increased, the operation conditions can be reduced, the material recovery can be realized, and it can also improve the efficiency of water pollutant degradation.

## 3 Water pollutant control using micro/nanorobots

### 3.1 Control principles and detection methods of water purification

#### 3.1.1 Control principles using micro/nanorobots

Continuous discharge of wastewater has significantly degraded soil and water quality, posing severe threats to organisms and ecosystems. Despite increasing evidence of worsening water pollution, research in this area remains limited (Bayabil et al., 2022). Along with the development of industry, more and more new pollutants gradually begin to accumulate and have negative ecological effects. These pollutants include polycyclic aromatic hydrocarbons, pharmaceuticals and personal care products, pesticides, phthalates, hormones, engineered nanomaterials, and perfluorinated compounds (Eumann and Schildbach, 2012; Gkika et al., 2022). Furthermore, these new pollutants, along with traditional pollutants such as metal ions and oil, synergistically interact with ecosystems, leading to more serious ecological disasters. For example, some studies have shown that the diffusion of antibiotics and heavy metals, among other factors, can result in bacteria mutations in wastewater. This, in turn, leads to the gradual increase of drug resistance and the development of new types of pathogenic bacteria, ultimately leading to infections with biological populations (Sambaza and Naicker, 2023). The spread of pollution in wastewater poses a threat that necessitates strategies to prevent further harm. This global public health issue demands immediate human action through wastewater treatment and water purification.

There are three primary steps involved in using micro/nanorobots for wastewater treatment and water purification, (i) capture, (ii) transfer, and (iii) degradation.

(i) The capture process typically involves electrostatic adsorption or chemical binding. However, as previously mentioned, the limited strength of the electrostatic adsorption effect may not always meet the requirements for effective use.
(ii) Transferring pollutants is sometimes not essential, as the development of micro/nanorobot technology is just beginning and cannot yet fully achieve real-time degradation of pollutants. Therefore, the proposed transfer is a compromise method to collect unprocessable pollutants and utilize other degradation methods. In the case of heavy metal pollution, the transfer of harmful elements out of the water body is also necessary.
(iii) The degradation process is the core of water pollution treatment, centered around a series of biochemical reactions that generate chemically active substances to break down pollutants into environmentally friendly substances. The most frequently employed active substance is Reactive Oxygen Species (ROS), which was first utilized in 1987 (Glaze et al., 1987), to facilitate the decomposition of organic matter into water and carbon dioxide. This approach simultaneously fulfills the fundamental requirement of avoiding secondary pollution, including the contamination of intermediate substances and the introduction of new pollutants during the treatment process. Numerous common mechanisms exist for pollutant degradation, with several of the more commonly used methods outlined here.
(i) Fenton reaction, involves the production of hydroxyl radicals from ferrous or ferric ions and hydrogen peroxide.
(ii) Fenton-like reaction, which is a similar process induced by other catalysts, e.g., Pt with H_2_O_2_ to produce hydroxyl radicals.
(iii) Heterogeneous photocatalysis, which means photons with energy greater than or equal to the band gap of the material irradiate the photocatalytic material (semiconductor or photosensitizer), generating electron-hole pairs that migrate to the surface of the photocatalyst, where they react with water and oxygen to produce ROS. ROS production is easier if a strong oxidizing agent, such as H_2_O_2_, is present in the process.

This is precisely why Fe_2_O_3_ nanoparticles are extensively employed in large quantities in the case above. Fe_2_O_3_ serves not only as an active semiconductor for visible light, enabling multiphase catalytic reactions under such illumination, but also as a catalyst for the Fenton reaction, which directly participates in the generation of active substances and promotes pollutant degradation. Additionally, Fe_2_O_3_ possesses magnetic properties, making it suitable for use as a magnetic module in micro/nanobots. As a result, it imparts the products with the capability of precise propulsion and magnetic recycling under magnetic control. This feature enhances the versatility of the products even further (Urso et al., 2023).

#### 3.1.2 Detection methods and integrated platforms

Detection methods play a crucial role in water purification experiments. Traditional water quality testing is mainly based on liquid or gas chromatography, combined with mass spectrometry. However, these methods demand substantial quantities of freezers and solvents for preparation (Wang et al., 2017), resulting in high operational costs. These methods are less effective in identifying trace contaminants like toxic ions and persistent organic pollutants. Different pollutants necessitate distinct observation and detection techniques. For instance, metal toxic ions are commonly detected using Atomic Absorption Spectroscopy (AAS) and Inductively Coupled Plasma Mass Spectrometry (ICP-MS), which offer high sensitivity but require bulky and expensive instruments unsuitable for on-site analysis. Dyes often rely on their distinct ultraviolet absorption peaks, while plastics are typically detected through Fourier Transform Infrared Reflection (FTIR), Raman spectrometry, and Mass Spectrometry (MS). Different sizes of pollution also require diverse equipment and methods. For micron-level pollution, initial detection is often accomplished through optical microscopy and scanning electron microscopy observations, whereas nanoscale pollution requires tools like transmission electron microscopy and nanoparticle tracking and analysis techniques (using diffraction imaging) for accurate observation. In the context of the micro/nanorobots discussed in this paper, fluorescence techniques and pulse voltammetry are the commonly employed detection methods (Rojas et al., 2016).

The advancement of experimental techniques in water refinement has spurred the exploration of integrated and functional experimental platforms. Zhao et al. (2023) developed a micro/nanorobot-based colorimetric detection platform, enabling rapid quantitative and high throughput qualitative colorimetry. The micromotors within the platform not only catalyze chemical reactions but can also transform into a rotor through magnetic drive, accelerating reactions via micro-stirring. Their platform system was made with 48 micro-orifices, which can perform up to 48 micro-droplet reactions simultaneously while rapidly catalyzing the target substances and displaying the corresponding colors for spectroscopic testing and analysis. The compact and portable integrated platform brings the traditional laboratory experience to water body testing, thereby significantly facilitating water quality assessment (Zhao et al., 2013).

**FIGURE 6:**
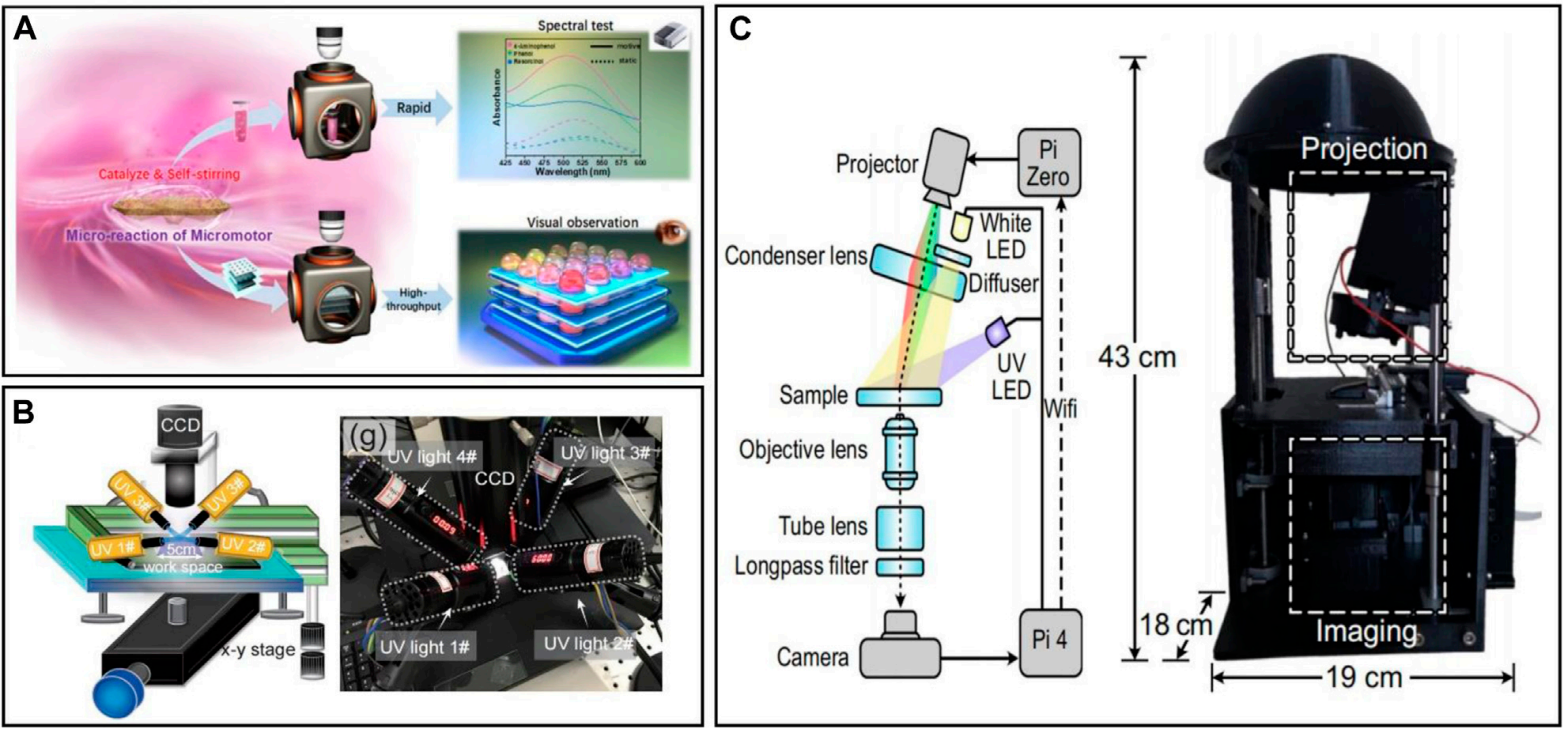
**(A)** Schematic illustrations of an integrated catalytic micromotor platform which enable efficient dual-mode colorimetric detection by self-stirring micro-reaction (Zhao et al., 2023). **(B)** Schematic illustration of the multi-light-fields based optical driving platform and a physical view of the multi-light-fields-based optical driving platform (Fan et al., 2023). **(C)** A picture of Dynamic Optical MicroEnvironment (DOME) with dimensions and the schematic illustration of the optical components (blue) and the wireless communication links (dashed lines) within DOME (Denniss et al., 2022).

Fan et al. (2023) faced the limitations of a single light source and developed a control platform based on multiple light field coupling, which realized the light-drive propulsion for both individual and collective micro/nanorobots, achieved by programming four light sources to emit in different directions. This platform served as a versatile tool for applying optical actuation to similar robots, meeting a range of purposes. Denniss et al. (2022) developed a low-cost, modular and open-source dynamic optical microenvironment, known as DOME, capable of inducing collective behaviors using light. This platform aimed to enhance the visualization of biologically-modified micro/nanorobots. The open-source modular design ensured high scalability and easy adaptation to new requirements, allowing the effective operation of different types of bio-modified micro/nanorobots by adjusting physicochemical parameters such as light source, microscope settings, ambient atmosphere, temperature, and so on. The DOME platform also offered the potential for future expansion of drive methods, the creation of a multi-drive observation setup, and even the integration of intelligent algorithms for analyzing single-unit behavior and group phenomena of the robots, along with monitoring physicochemical parameters. The images and the illustrations of the above platforms this paper mentioned above is shown in Figure 6.

### 3.2 Latest research on water pollution control using micro/nanorobots

#### 3.2.1 Removal of micro/nanoplastics

Plastics are composed of long chains of polymers, with covalent bonds connecting the monomers. Due to their low cost and good performance, they are widely used in industry and daily life. However, the non-biodegradability of certain plastics has resulted in their accumulation within the ecosystem. Moreover, plastics not only serve as environmental pollutants themselves but also trigger a series of ecological hazards. Micro/nanoplastics encompass a range of plastic particles with dimensions smaller than 5 mm. Owing to their small size and large surface area, microplastics readily absorb pollutants and offer substrates for the growth of microorganisms like bacteria. Additionally, they attract diverse pollutants through adsorption, culminating in heightened toxicity. Generally, nanoplastics are prone to suspension and dispersion due to water turbulence. Their higher surface-volume ratio results in enhanced adsorption of microorganisms and contaminants, facilitating deeper penetration into biological tissues. Recently, micro/nanoplastics have been found in various organisms. Shrimp ingest these particles while feeding on decaying material or plankton, causing issues such as appetite loss and tissue deformation (Urso et al., 2023). Research in 2022 measured the mass concentration of plastic polymer components in human blood for the first time, showing the absorption of plastic particles into the bloodstream (Leslie et al., 2022). In addition, micro/nanoplastic particles have been found in different plant and animal species, including fish and algae (Ribeiro et al., 2023).

Micro/nanorobots use various methods for capturing and degrading micro/nano plastics. The three primary capture methods include i) electrostatic interaction, ii) electrophoretic attraction and iii) adhesion. i) Electrostatic interactions require surface charge programming, typically achieved by pH adjustment. For example, TiO_2_ becomes positively charged in acidic conditions and negatively charged in alkaline surroundings. However, precise pH control can be challenging even in confined spaces. ii) Electrophoretic attraction relies on creating chemical gradients around operating micro/nanorobots, facilitating the capture of micro/nanoplastics. iii) Adhesion is achieved by mimicking DOPA proteins secreted by mussels, through a structurally similar design of another protein PDA, which can be used as coatings for microplastic adhesion. After capturing the micro/nanoplastics, the micro/nanorobot completes their oxidation through several methods. This includes breaking the chemical bonds of polymers, photocatalytic degradation, and enzymatic degradation. For example, the Fenton reaction can be employed to break the polymer bonds, while photo-active materials generate electron-hole pairs under light, initiating chemical reactions with water and other substances. This generates reactive oxygen species (ROS), achieving photocatalytic degradation. Alternatively, enzymes can be directly used for decomposition.

**FIGURE 7:**
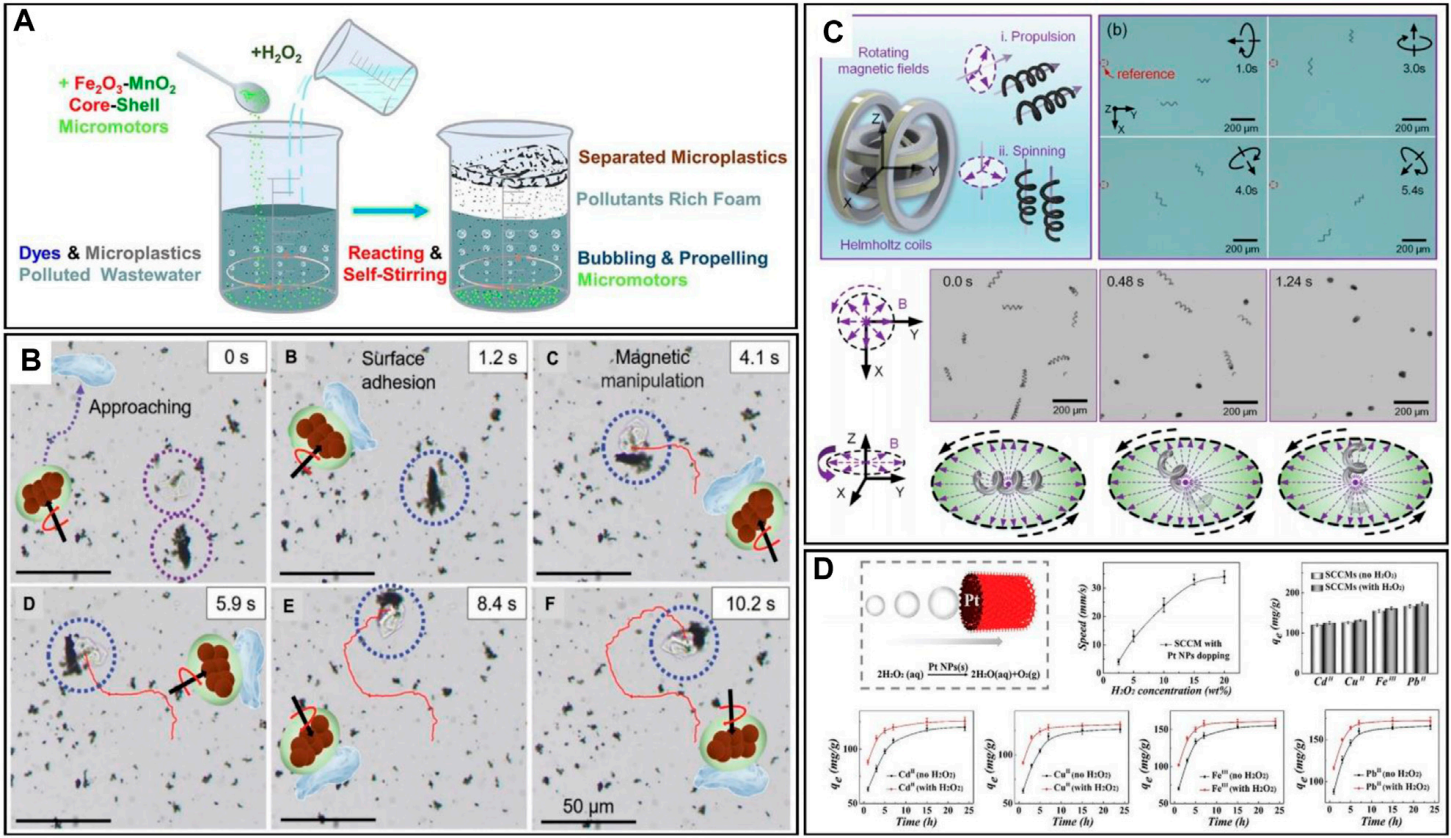
**(A)** Schematic illustration of adsorptive bubble separations for dissolved pollutants and microplastic removals (Ye et al., 2021). **(B)** Time-lapse of the microplastic removal process using PDA@Fe_3_O_4_ MagRobots. The image shows approaching, Surface adhesion and movement with the assistance of a transversal rotating magnetic field (Zhou et al., 2021b). **(C)** Schematics illustration of the BMHMs’ propulsion and spinning, and time-lapse images of the BMHMs in the movement mode of directional propulsion, stand erect and spin around (Gong et al., 2021). **(D)** Schematic diagram of the self-propelled SCCM, and the plot of the movement speed of the SCCM in different concentrations of H_2_O_2_. The diagram also gives a comparison of the effect of the presence or absence of H_2_O_2_ on adsorption in a static environment, and shows adsorption of different soluble metal ions by the SCCM under different conditions (Shang et al., 2022).

In recent years, a notable study on plastic treatment involves the use of two bubble-propelled micro/nanorobots conducted by Ye et al. (2021). They used two motors, Fe_3_O_4_-MnO_2_ and Fe_2_O_3_-MnO_2_, for microplastic decomposition, demonstrating that the Fe_2_O_3_-MnO_2_ microrobot effectively eliminates microplastics suspended in aqueous solution through the synergistic action of catalytic degradation, surface adsorption and adsorption bubble separation mechanisms (Figure 7A). Remarkably, adsorption bubble separation led to over 10% removal of suspended microplastics from contaminated water within a span of 2 h. The study also clarified the main contributions of different remediation mechanisms in pollutant removal. Zhou et al. (2021b) prepared adherent polydopamine PDA@Fe_3_O_4_ magnetic microrobots called “MagRobots”, by modeling the basic properties of marine mussel adhesion. Microplastic enzymatic degradation was achieved by loading lipase onto the surface of PDA@Fe_3_O_4_-MagRobots. Leveraging magnetic actuation, these robots exhibited good kinematic abilities, enabling them to navigate predefined path and precisely target and remove microplastics at a specific point. The trajectories of the robot guided by magnetic field can be seen in Figure 7B.

#### 3.2.2 Removal of heavy ions

Heavy metal ions, primarily consisting of elements with relative atomic masses ranging from 63 to 200, are mainly transition metals such as Ni, Hg, Pb, Cu, Zn and Cd. These metals are difficult to degrade when dissolved in water, thus they are prevalent pollutants with the capacity to accumulate in sedimentary layers within soil, plants and animals, posing a persistent threat to public health. This includes potential risks such as myocardial infarction and lung cancer. The conventional approach to removing heavy metal ions is electrocoagulation, which offers a practical solution for eliminating heavy metals and other pollutants from water. This method is particularly attractive due to the small size of sludge generated during treatment, but its widespread implementation in many facilities is limited by the high costs involved (Aguiar et al., 2023). Furthermore, hexavalent ferrate has been used to remove various metal ions, including Pb, Cd, Fe, Zn, Mn, Cu, As, Co, Ni, and Al. Research findings indicate that hexavalent ferrate can be used as a multipurpose chemical for the effective removal of toxic metal ions. However, its efficiency in removing certain harmful metals is limited, and it can even have adverse effects on water quality. Practical tests involving hexavalent ferrate have been carried out, but the outcomes have often been unsatisfactory (Dong et al., 2019). Over the past years, micro/nanorobots have emerged as a promising approach for treating metal ions, attracting attention for their fast and thorough clean-up capabilities.

**FIGURE 8:**
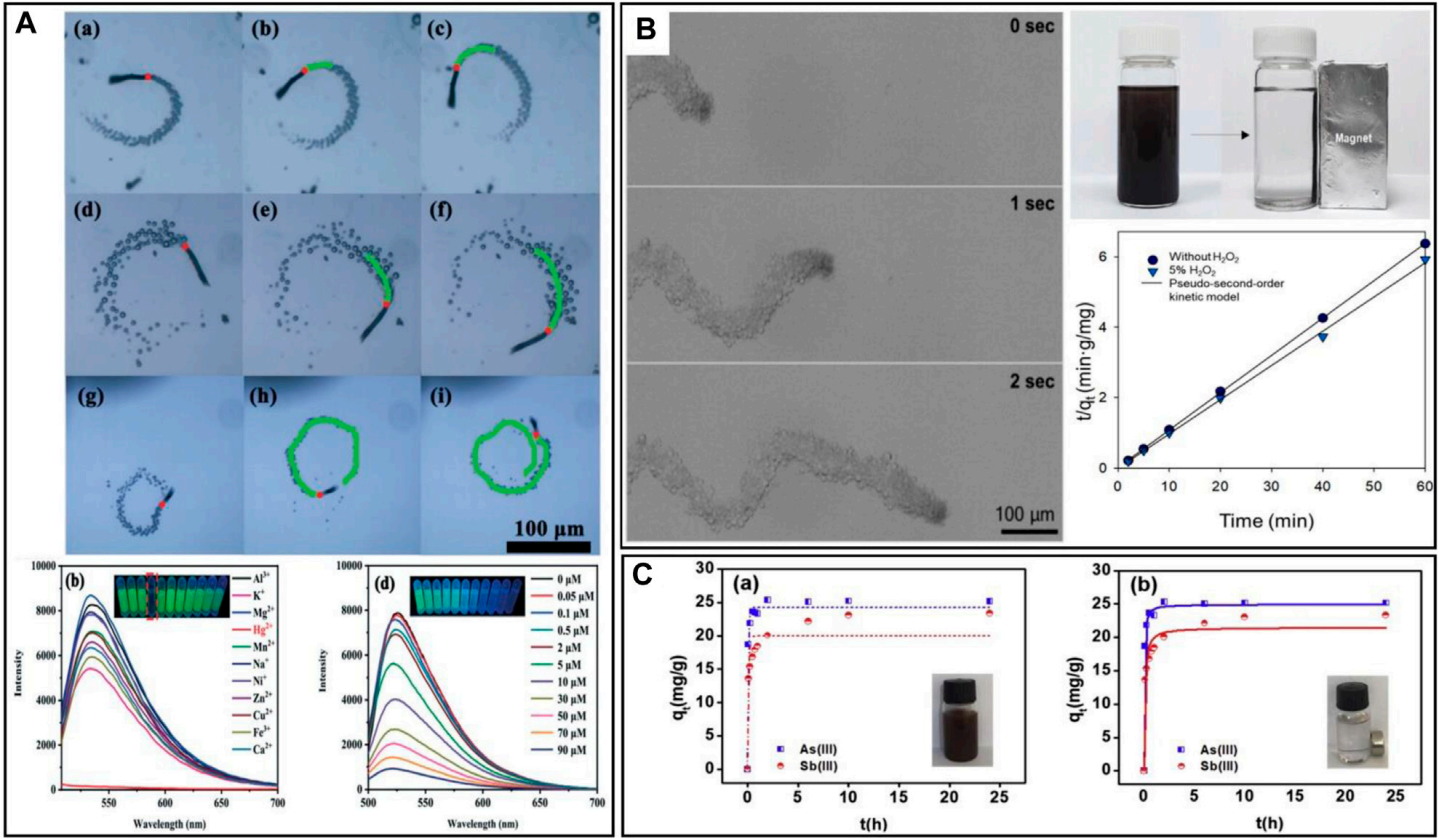
**(A)** One second Time-lapse images of the AO–Mn_2_O_3_/γ-AlO&IJlig;OH micromotor in H_2_O_2_ solutions with different concentrations of 1%, 2%, and 3%. The images below are the fluorescence effects of the measurement of different metal ions, and fluorescence effects of the measurement of different concentrations of Hg^2+^ (Li et al., 2021). **(B)** Time-lapse images of the MIMS/Pt motors in the environment of 10 wt% H_2_O_2_ with 0.5 wt% Triton-X100. The image of the robot recycling, shows that a magnet will do the trick. The last diagram shows the Cs removal process of the MIMS/Pt under varying conditions (Park et al., 2021). **(C)** Adsorption performance of As^3+^ and Sb^3+^ in the singular system on Fe_3_O_4_@TA@UiO-66 (Qi et al., 2019).

(i) Lead ions. Lead ions are among the most abundant heavy metals in the Earth’s crust and are widely used in industry. Unfortunately, the widespread utilization of lead has led to its prevalence as an environmental pollutant. Being a toxic heavy metal, lead is a neurotoxic metal with no metabolic benefits, easily absorbed by the body, non-biodegradable by living organisms, and extremely difficult to discharge (Wu et al., 2023). Addressing this issue, Gong et al. (2021) designed Fe_3_O_4_-MnO_2_ biohybrid magnetic helical microrobots (BMHMs) based on Spirulina cells. Actuated by the rotating magnetic fields, these robots can accomplish spinning around three orthogonal axes (demonstrated in Figure 7C). These microrobots demonstrated a remarkable ability to remove Pb^2+^ from wastewater and achieved an adsorption efficiency exceeding 95%. It is noteworthy that the BMHMs can also be recycled after simple regeneration.
(ii) Copper ions. Copper is usually recognized as a highly hazardous heavy metal (Rahman et al., 2023). While it is essential for vital human functions and physical wellbeing, excessive copper intake can result in acute gastrointestinal symptoms, liver enzyme systems inactivation, and even movement disorders. Global copper pollution has been on the rise in the aquatic environment and has been recognized as a significant source of heavy metal contaminants due to health risks (Liu et al., 2023). In response to this concern, Shang et al. (2022) developed micro/nanorobots known as SCCMs using responsive hydrogels of carboxymethyl chitosan (CMC) and polyacrylamide (PAM) labelled with platinum nanoparticles (PtNPs). These robots demonstrated the ability to treat a wide range of heavy metal ions (see details in Figure 7D), including Cu^2+^. The SCCMs robots exhibited a self-reporting feature, making the adsorption/desorption process visible to the naked eye through observable colour change.
(iii) Mercury, found in substances like mercury thermometers, is one of the most common and accessible heavy metals. However, it poses significant bio-toxicity, especially in the form of free mercury ions, which can lead to mercury poisoning upon entering the human body. In response, Li et al. (2021) developed a new fluorescent micromotor propelled by the asymmetric decomposition of hydrogen peroxide, with Mn_2_O_3_ serving as the catalyst. The fluorescence effect of the micromotor showed good selectivity and sensitivity in detecting Hg^2+^ (Figure 8C), enabling the detection of mercury ions even at low concentrations.
(iv) Radioactive ions, specifically dissolved radionuclides, exhibit mobility within the natural environment. If not appropriately treated, they can enter the aquatic environment, such as rivers and groundwater. This increases the risk of biological exposure to radioactive radiation. In contrast to other pollutants, radioactive contaminants have extremely long half-lives and are more insidious and potent toxins that can remain for extended periods (Ma et al., 2023). In this regard, Park et al. (2021) tackled the removal of radioactive ^137^Cs from contaminated solutions using a clay Janus microsphere micro/nanorobot (Figure 8B). Their robots have outstanding repulsive magnetism and motion effects, by incorporating a magnetic module, the micro/nanorobot could precisely locate radioactive sources under an applied magnetic field in a liquid environment containing competing ions, and the results demonstrated that the robot could remove more than 98.6% of ^137^Cs from an aqueous medium containing potassium, sodium, calcium, and magnesium ions.
(v) Arsenic. Although arsenic is not classified as a heavy metal, its contamination is often treated as such. Arsenic poses a high level of toxicity to all forms of life. The element has been designated as a Group I human carcinogen with the highest classification by the World Health Organization (Nicomel et al., 2016). Traditional techniques for removing arsenic include oxidation, coagulation-flocculation and membrane methods, but these approaches are currently not deemed sufficiently practical due to cost and process limitations (Dilpazeer et al., 2023). Addressing this issue, a novel magnetic core-shell microsphere named Fe_3_O_4_@TA@UiO-66 was developed by Qi et al. (2019). This microsphere has the capability to remove both As^3+^ and Sb^3+^ from wastewater. The adsorption experiments carried out have demonstrated the high adsorption capacity of the magnetic Fe_3_O_4_@TA@UiO-66 for As^3+^ and Sb^3+^ (Figure 8C). Moreover, it can be rapidly separated from the aqueous medium within 2 min after treatment and the composite exhibited effective removal performance for As^3+^ and Sb^3+^ across a wide range of solution chemical conditions.

#### 3.2.3 Removal of oil

Environmental pollution caused by oily substances, like oil, primarily results from human activities, particularly offshore oil spills and wastewater discharges from industrial platforms. Oily substances not only induce soil toxicity but can also infiltrate the food chain via water sources, causing harm to ecosystems. The potent accumulative impact of oil pollution can easily lead to the phenomenon of bioaccumulation in organisms.

**FIGURE 9:**
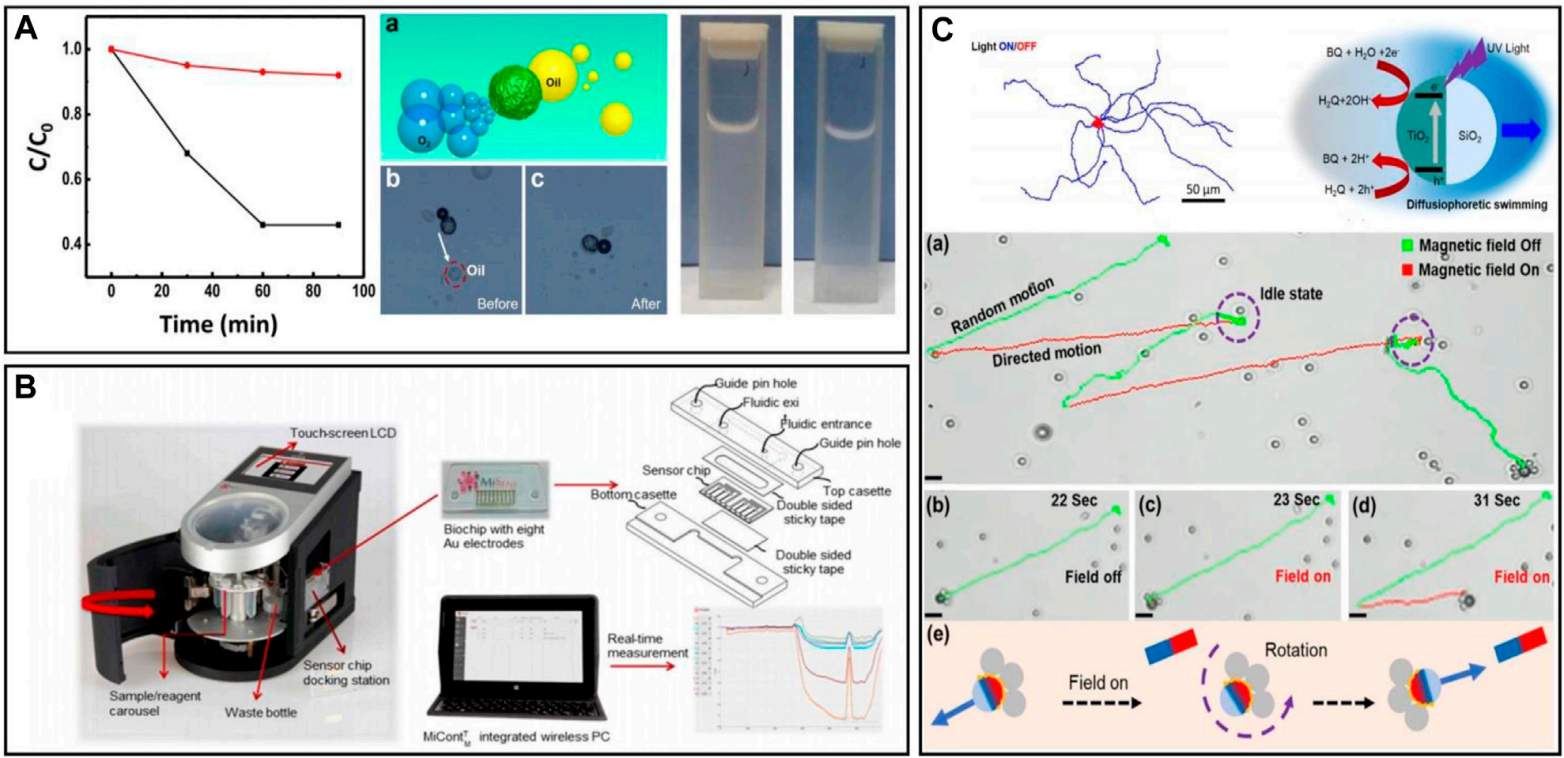
**(A)** Effectiveness curve and visual representation of the degreasing by the walnut-like micromotor, the red curve represents the stationary counterpart, and the images of the process of the capture of the oil by the walnut-like micromotor (Wang et al., 2019). **(B)** Schematic illustration of microfluidic-based electrochemical biosensor (Wang et al., 2022). **(C)** Trajectories of microrobots under light with light on (blue) and off (red), and the schematics illustration of the reaction. The photos below show the total and detail of the movement of PEDOT/MnO_2_@Ag micromotors, and the motion mechanism guided by a magnet (Debata et al., 2022).

Addressing this issue, Wang et al. (2019) developed a multi-responsive walnut-shaped microrobot that achieved kinetic and magnetic responses through catalase and ferric tetroxide, generating motion via bubble formation in a fuelled environment. The hydrophobic PCL polymer component within the microrobot enabled strong adhesion upon contact with oil droplets, facilitating the collection of oil droplets from the solution (Figure 9A). The removal of oil droplets and energy reuse can be achieved through magnetic field separation. Similarly, Ma and Wang. (2019) introduced a microrobot based on a magnetically propelled superhydrophobic sponge, which effectively captured chloroform, methylene chloride, and toluene with high capacity. The sponge microrobot enabled precise motion control and selective separation simultaneously. Even after five regeneration and reuse cycles, the sponge robots maintained their high absorbency and recycling capacity. Wang et al. (2022) introduced an efficient robotic platform for pollutant adsorption (Figure 9B). The system involved a flapping-wing micro-plane for long-distance travel and a low-cost multifunctional Janus microrobot for pollutant purification. During operation, the flapping-wing aircraft flied over the targeted water body, releasing the microrobot. Comprising silicon microspheres, a nickel layer and a hydrophobic coating, the robot is designed to absorb oil and treat organic pollutants. Controlled by a rotating magnetic field, the manipulable microrobot can navigate the water environment, continuously searching for oil droplets.

#### 3.2.4 Removal of microorganism

Waterborne diseases from pathogenic microorganisms pose a severe global health threat. Globally, approximately 1.6 million annual deaths are due to biological contaminants in water, like bacteria, viruses, protozoa and worms. These microorganisms can enter the human body to cause health risks through direct consumption of contaminated water (Kumar et al., 2019). *E. coli*, common in freshwater, is antibiotic-resistant and can cause serious illnesses like haemorrhagic colitis and haemolytic uremic syndrome through the production of verodoxin (Siri et al., 2023). Initial techniques to eliminate harmful organisms in water included antibiotics, enzymes, metal ions, *etc.*, or UV light, chlorine compounds, and ozone. However, these methods generate toxic by-products.

Debata et al. (2022) developed a structurally simple Ni-Au/TiO_2_-SiO_2_ Janus robot for safe capture of *E. coli*. Fuelled by hydroquinone/benzoquinone, the speed of the robot could be adjusted by changing the UV intensity (Figure 9C). The microrobot captured and transported silicon particles and *E. coli* through a Ni-Au coating, where the introduction of Ni allowed the robot to be driven by a magnetic field. *E. coli*’s safety during capture was confirmed via live/dead fluorescent dye test. Liu et al. (2020) proposed PEDOT/MnO_2_@Ag microrobots for microbial killing based on the excellent bactericidal ability of silver ions. The synergistic catalytic action of MnO_2_ and Ag facilitated rapid swimming in low H_2_O_2_ levels. Compared to a 0.2% hydrogen peroxide, *E. coli* death increased by about 14%, benefiting from the combined effect of the mass transfer due to the continuous motion of the microrobot and the dynamic release of silver ions.

#### 3.2.5 Removal of harmful organics

In recent years, there have been reports of using micro/nanorobots for the removal of other organics present in water treatment processes, such as dyes, pesticides, psychoactive drugs, phenol and therapeutic drugs.

**FIGURE 10:**
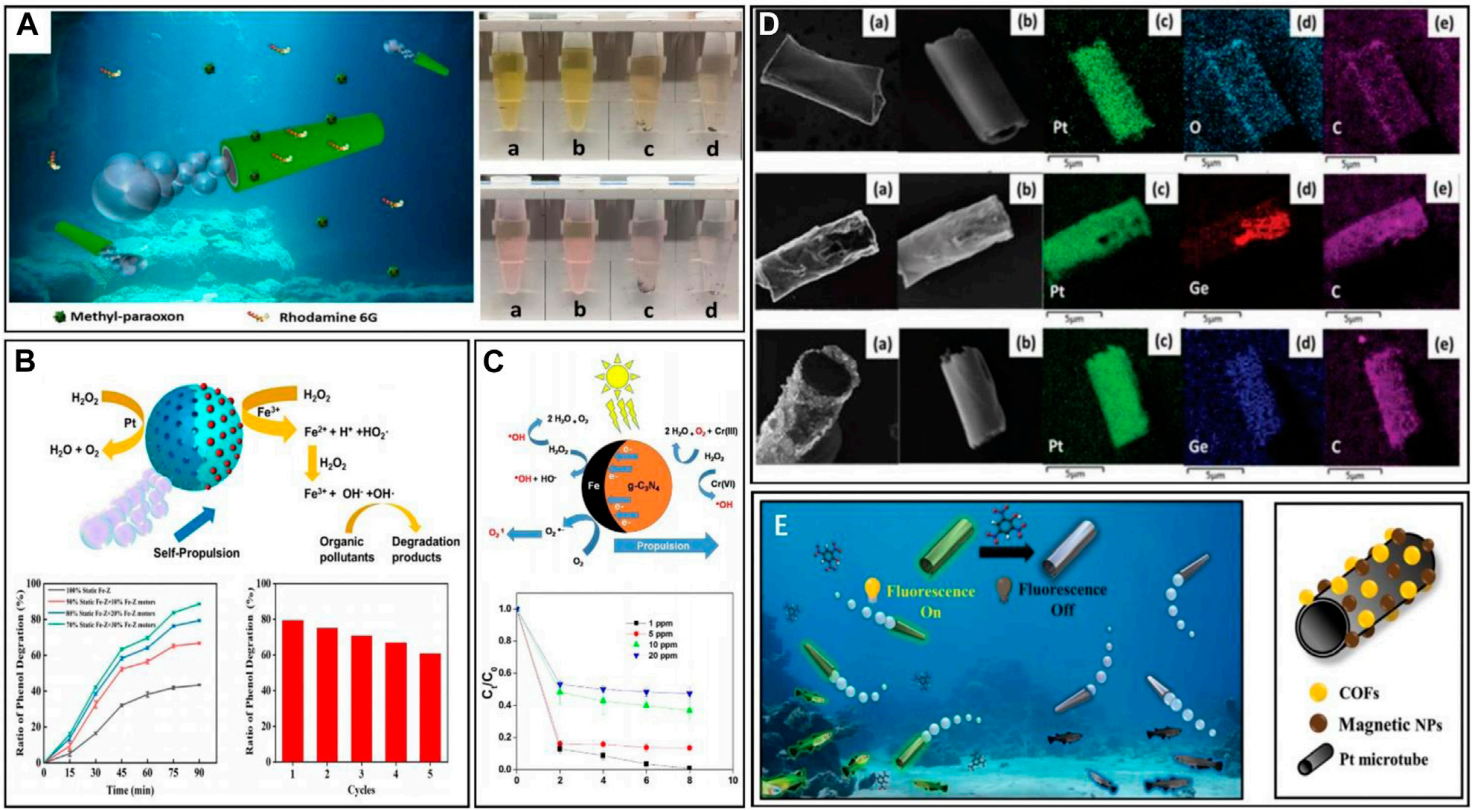
**(A)** Schematics illustration of the self-propulsion and pollutants purification by artificial multilayered microrocket, and diagrams of the removal of methyl-paraoxon and rhodamine 6G by artificial multilayered microrocket (Li et al., 2015). **(B)** Schematics illustration of the degradation of organic pollutants by Fe-zeolite micromotors. The diagram shows the effect of initial conditions on phenol degradation and the degradation efficiency in the reuse of the microrocket (Ma et al., 2020). **(C)** Schematics illustration of the motion of the microrobot and the generation of ROS, and the effect of initial SMX concentration on degradation (Rayaroth et al., 2021). **(D)** Energy-dispersive spectroscopy (EDS) elemental mappings of bare GO-Pt, 2D-Me-Ge modified and 2D-Ph-Ge modified microrobots respectively (Moric et al., 2020). **(E)** Fluorescent mechanism of the magnetic COF-functionalized micromotor, and the schematic diagram of the structure of the magnetic COF-functionalized micromotor (Wang et al., 2021).

(i) Dyes. Textile wastewater generally contains a variety of synthetic dyes, including azo dyes, indigo dyes, triphenylmethane dyes, anthraquinone dyes and aromatic methane dyes. These dyes exhibit specific traits such as resistance to light, acid and alkali treatments, which can pose potential health risks like carcinogenicity and teratogenicity (Deng et al., 2018). In this regard, Uygun et al. (2021) designed a self-propelled microrobot powered by laccase, which effectively degraded various dyes. Their micro motors successfully removed a wide range of dyes, including acid red, reactive brown 10, alkali blue 6B, and bright blue 6, with dye concentration reduction ranging from 76% to 94%, proving the overall dye removal ability of the robot. Li et al. (2015) designed a multilayer microrocket and investigated its absorption behavior for pollutant purification. Their study proved that the H_2_O_2_ fuel concentration has a positive effect on the motion of the microrockets (Figure 10A), and by controlling the direction of the applied magnetic field, the motion of the microrockets in any required direction can be controlled. In addition, the motion of the multilayered microrockets enhanced the fluid dynamics and improved the removal of methyl-paraoxon and rhodamine 6G and so the water purification efficiency.
(ii) Pesticides. The use of pesticides has tripled in the last 50 years (Wang et al., 2022). Widespread conventional pesticide use has become ecotoxic to plants, animals, and microorganisms, and emerging nano-pesticides have been used without proper environmental assessment (Ale et al., 2023). In this regard, Song et al. (2023) designed a micro/nanorobot using magnetotropic bacteria (Magnetospirillum AMB-1) along with organic matter to effectively remove chlorpyrifos (a widely used organophosphate pesticide in agriculture) from various aqueous solutions. The robot can be precisely controlled under a magnetic field. When subjected to a clockwise magnetic field, the swarm exhibits circular swarming behavior. The micro-mixing during this process improved pesticide removal efficiency. The controllable magnet AMB-1 can be propelled under a magnetic field to efficiently remove pesticides dynamically. Authors suggest a potential approach for practical application by placing permanent magnets on water pipes in treatment plants to operate a swarm of machines inside pipes for toxin treatment.
(iii) Psychoactive drugs. Various psychoactive compounds (e.g., carbamazepine, pesticides, *etc.*) have accumulated in the environment, directly affecting freshwater systems, soils, and organisms. These compounds are found in plants through soil accumulation and in animals through the food chain. Exotic psychoactive substances have even been discovered in fish brains (Vejvodova et al., 2023). Pacheco et al. (2022) developed a PEI@PCL/Fe_3_O_4_ microrobot to remove nitroxide Sulphur ions (neurotoxic agents within pesticides) from real water samples. In experiments, the robot effectively removed the target from complex water samples such as tap water, when subjected to a magnetic field. The robot achieved about 60% removal of the nerve agent in a short time. Notably, the researchers used real water samples instead of idealized samples, enhancing the credibility of their results. To us, this work provides a reference sample for the large-scale removal of nerve agents from water bodies.
(iv) Phenol. Due to its high toxicity and limited biodegradability, phenol can act as a pollutant even at very low concentrations. It is toxic, carcinogenic, mutagenic and teratogenic. Several methods have been developed for the removal of phenolic compounds from wastewater, including common chemisorption and oxidation techniques, which however, come with high energy and maintenance costs (Saputera et al., 2021). Recently, Ma et al. (2020) designed a micromotor for efficient phenol degradation using a Fenton-like reaction on iron zeolite (Figure 10B). The micromotor featured a porous structure with enhanced catalytic properties. The process employed an asymmetric Pt layer to catalyze the decomposition of H_2_O_2_, enabling the self-propulsion of the robot. The self-propelled motion, along with the formation of bubbles, enhanced fluid mixing and improved the degradation efficiency. The iron zeolite micromotor can be collected and reused to prevent secondary pollution and waste.
  (v)Therapeutic drugs. The most commonly found drugs in groundwater include anti-inflammatory medications such as diclofenac, paracetamol (PTM) and ibuprofen. Prolonged exposure to these compounds can have harmful effects on biota, such as gill changes in fish and kidney problems in birds. Antibiotics are another group of drugs widely present, which greatly favor the emergence of drug-resistant bacteria and have been identified as significant water pollutants (Sacco et al., 2023). Rayaroth et al. (2021) developed a Janus Fe/C_3_N_4_ microrobot driven by a chromate-hydrogen peroxide (Cr^6+^/H_2_O_2_) redox system, with its kinematic attributes analyzed. The findings indicated that Cr^6+^ alone cannot effectively drive the Fe/C_3_N_4_-based microrobot, and it was the Cr^6+^/H_2_O_2_ redox system generating oxygen bubbles that significantly propel the C_3_N_4_ robot. Here the formation of active substance ROS was confirmed by electron spin resonance experiments to effectively degrade sulfamethoxazole (SMX) (Figure 10C). Ye et al. (2022) designed a Fe-MnO_2_ microrobot to remove tetracycline antibiotic contaminants. Their simple design, easy fabrication, good kinematic performance, magnetic recovery capability, and excellent decontamination effectiveness across a wide pH range served as a valuable reference for the degradation of antibiotics in water.

### 3.3 Water monitoring

In the previous section, this paper focused on water treatment and purification. However, complete pollution clean-up cannot be quickly achievable, and water bodies face potential secondary pollution risks. Hence, long-term monitoring of water quality is necessary. Water quality monitoring entails identifying and quantifying pollutants, serving as an important tool for decision-making and improving water quality. Furthermore, traditional techniques for monitoring water are often costly, requiring skilled professionals and complex equipment, and in many cases do not allow for direct analyses and immediate results in polluted areas. Presently, the emphasis in water monitoring technology lies in developing low-cost, convenient and user-friendly devices and sensors, aiming to make significant strides in this domain (Silva et al., 2022).

Traditional methods for real-time water monitoring involve utilizing various spectroscopic detection techniques and molecular methods (Zolkefli et al., 2020). These instruments are not only cumbersome but also inevitably introduce data errors for water bodies that cannot be preserved over extended periods in water quality classification. *In situ* measurements help to reduce costs by eliminating the need for sampling, sample preservation, transportation and laboratory analyses, which is where the advantage of micro/nanorobots lies. Due to current technological constraints, micro/nanorobot systems and platforms are not yet capable of full-scale water quality monitoring, only water quality testing. However, the use of micro/nanorobot systems for water quality analysis offers the advantages of cost-effectiveness and immediate on-site results. These systems can already serve as an alternative means of gathering data to improve water quality. In other words, while micro-nano robotic systems cannot entirely replace the water monitoring process, they can effectively substitute for several important testing stages.

In addition to the colorimetric method mentioned above, fluorescence methods offer an alternative approach to water monitoring. Fluorescence techniques address two main challenges. Firstly, integrating all target substances into a single micro/nanorobot platform remains unfeasible, which means that there is a possibility of using multiple robots simultaneously in the water body, necessitating rapid identification using fluorescence methods. Moric et al. (2020) have developed highly stable fluorescent markers based on chemically modified 2D germanium compounds, available in a variety of different marker colors such as blue, violet and red, facilitating the tracking of specific robots within intricate swarms of micromachines. The efficacy of these markers was demonstrated through experiments, where robots carrying target substances were labelled with blue markers, while naked robots were marked with a red substance (Figure 10D). These robots exhibited strong and distinct fluorescence signals, thus offering a potential avenue for imaging. Secondly, the water column is cluttered with contaminants, demanding distinct responses for different contaminants. Wang et al. (2021) demonstrated a nanorobot equipped with a fluorescent ‘switch’ for detecting explosive substances in water (Figure 10E), and by combining the change of fluorescence with the work of Maric et al. (2020), differentiated responses to different substances can be achieved.

Based on the method of water monitoring using micro/nanorobots, there is a requirement to develop and design evaluation metrics and engineering systems for micro/nanorobot-assisted water monitoring systems. The existing metric employed for water quality assessment is the Water Quality Index (WQI), which encompasses various physicochemical parameters of water such as dissolved oxygen, total bacterial flora, pH, temperature, nitrogen, phosphorus, and turbidity (Shen et al., 2016). The WQI, which first appeared in 1960 (Horton’s Index), enables the quantification of water quality in diverse contexts, including recreational, irrigation, and public water supply. Therefore, developing WQI indicators based on micro/nanorobots presents a significant avenue for integrating these systems into long-term monitoring of water quality. This involves identifying pertinent physicochemical parameters with superior performance for micro/nanorobots, establishing parameter ranges through extensive experimentation, and ultimately setting industry standards.

Finally, the integration of micro/nanorobots with emerging technologies can also be accelerated. The main technologies in progress encompass colorimetric methods or electrochemical sensors for assessing water bodies like drinking water, rivers and lakes. Nevertheless, there are well-established micro/nano-robotic systems, technologies and approaches that can simplify and improve existing water monitoring technologies while also reducing costs. Additionally, the integration of water monitoring links with the Internet can also accelerate data collection abilities, which is relevant for real-time, online water quality monitoring.

## 4 Conclusion and future perspective

This paper reviews the progress made in the past 5 years regarding micro/nanorobots in addressing water pollution. Although micro/nanorobots have exhibited several benefits, including high efficiency, precise motion control, targeted pollutant removal, and recyclability, challenges remain to be addressed within this field.

(i) Costly

Apart from employing basic chemical methods like hydrothermal methods for manufacturing micro/nanorobots, several of these approaches can be costly, potentially impacting the feasibility of mass production and industrial implementation. The incorporation of precious metals like platinum, gold, and silver in micro/nanorobots also raises material expenses. Reducing the utilization of precious metals to enhance cost-effectiveness has become a mainstream development in the industry.

(ii) Capture without degradation

According to the literature collected and analyzed, it is evident that not all micro/nanorobots can successfully attain the ultimate objective of pollutant degradation. Presently, numerous robots are limited to merely removing contaminants from the water column, which is mainly caused by insufficient available oxidation techniques and the absence of integration of functional modules within the robot.

(iii) Utility in open water bodies

The direct decontamination of open water bodies using micro/nanorobots remains unrealized. On one hand, the stability of robot movement is not yet high enough to resist the various unpredictable factors present in the natural environment. On the other hand, the mechanisms of capture and degradation, along with the excessively large movement space may not be suitable for water bodies exposed to nature. It has been suggested that the application of micro/nanorobots for water purification should not be aimed at open water bodies, but rather confined within specific containment units, which would serve as the basic modules for water purification, and then can be replicated to achieve industrialization and factorization (Urso et al., 2023).

(iv) Idealization Experiments

The current laboratory-based advancements often remain highly theoretical, with limited experimentation involving actual water samples. Meanwhile, target substances are often overly idealized, such as replacing dyes with methylene blue and rhodamine B, or employing *E. coli* as a proxy for harmful bacteria. These factors contribute to a disconnection between the existing outcomes and practical real-world applications.

(v) Secondary Pollution

Micro/nanorobots could potentially contribute to secondary water pollution. Firstly, the biochemical reactions occurring during the operation of these robots must be thoroughly assessed to prevent the generation of potentially bio-toxic substances. Additionally, the widespread deployment of micro/nanorobots may lead to their accumulation in the ecosystem, transforming them into environmental pollutants. To address this concern, the use of biodegradable materials should be prioritized in the design of micro/nanorobots.

(vi) Better evaluation indexes are needed

Currently, there is a lack of standard evaluation indexes for the experiments on water pollution treatment of micro/nanorobots. For example, the pollutant removal effect, the removal time needed, *etc.* Most of the indicators used now are based on the experience of the experimenters. Scholars, including Mihail N. Popescu, have recognized this issue, proposing qualitative analyses of small samples over quantitative analyses in resource-poor environments (Popescu and Gaspar, 2023). In the future, the establishment of complete engineering metrics is essential to facilitate the widespread adoption of micro/nanorobots for addressing water pollution challenges.

(vii) Further performance improvements

While this paper frequently employs the term “micro/nanorobots”, it is important to note that the existing research may not fully encompass the capabilities of a genuine “robot”. Until now, the terms “micro/nano-swimmers” or “micro/nano-motors” offer a more accurate depiction of these entities. Micro/nano-robotic systems still need further exploration of diverse propulsion methods, in-depth studies on multifunctional integration, exploration of potential collective effects, and addressing important issues such as robot signal processing, self-repair, and human-robot interaction. These efforts are imperative for comprehensively improving performance and expediting the industrialization of this field.
